# *Clostridium difficile* Spore-Macrophage Interactions: Spore Survival

**DOI:** 10.1371/journal.pone.0043635

**Published:** 2012-08-27

**Authors:** Daniel Paredes-Sabja, Glenda Cofre-Araneda, Christian Brito-Silva, Marjorie Pizarro-Guajardo, Mahfuzur R. Sarker

**Affiliations:** 1 Laboratorio de Mecanismos de Patogénesis Bacteriana, Departamento de Ciencias Biológicas, Facultad de Ciencias Biológicas, Universidad Andres Bello, Santiago, Chile; 2 Department of Biomedical Sciences, Oregon State University, Corvallis, Oregon, United States of America; 3 Department of Microbiology, Oregon State University, Corvallis, Oregon, United States of America; Indian Institute of Science, India

## Abstract

**Background:**

*Clostridium difficile* is the main cause of nosocomial infections including antibiotic associated diarrhea, pseudomembranous colitis and toxic megacolon. During the course of *Clostridium difficile* infections (CDI), *C. difficile* undergoes sporulation and releases spores to the colonic environment. The elevated relapse rates of CDI suggest that *C. difficile* spores has a mechanism(s) to efficiently persist in the host colonic environment.

**Methodology/Principal Findings:**

In this work, we provide evidence that *C. difficile* spores are well suited to survive the host’s innate immune system. Electron microscopy results show that *C. difficile* spores are recognized by discrete patchy regions on the surface of macrophage Raw 264.7 cells, and phagocytosis was actin polymerization dependent. Fluorescence microscopy results show that >80% of Raw 264.7 cells had at least one *C. difficile* spore adhered, and that ∼60% of *C. difficile* spores were phagocytosed by Raw 264.7 cells. Strikingly, presence of complement decreased Raw 264.7 cells’ ability to phagocytose *C. difficile* spores. Due to the ability of *C. difficile* spores to remain dormant inside Raw 264.7 cells, they were able to survive up to 72 h of macrophage infection. Interestingly, transmission electron micrographs showed interactions between the surface proteins of *C. difficile* spores and the phagosome membrane of Raw 264.7 cells. In addition, infection of Raw 264.7 cells with *C. difficile* spores for 48 h produced significant Raw 264.7 cell death as demonstrated by trypan blue assay, and nuclei staining by ethidium homodimer-1.

**Conclusions/Significance:**

These results demonstrate that despite efficient recognition and phagocytosis of *C. difficile* spores by Raw 264.7 cells, spores remain dormant and are able to survive and produce cytotoxic effects on Raw 264.7 cells.

## Introduction

*Clostridium difficile* is a Gram-positive, anaerobic bacterial pathogen, responsible for ∼20% of antibiotic-associated diarrheas, pseudomembranous colitis and toxic megacolon [1], [2]. Onset of *Clostridium difficile* infections (CDI) typically occur during or after antibiotic treatment of hospitalized patients depending on whether the infected *C. difficile* isolate exhibits resistance to the antibiotic being administered [2]. Antibiotics disrupt the normal colonic flora, which normally suppresses *C. difficile* growth, therefore allowing *C. difficile* to colonize empty niches and secrete two major toxins, TcdA and TcdB producing massive intestinal epithelium damage [2]. In addition, both toxins trigger the release of various cytokines and chemokines that lead to an intensive immune response resulting in the recruitment of neutrophils and macrophages from the systemic system [2]. During the onset of CDI, *C. difficile* begins a sporulation cycle in the colon [1], [3] leading to persistence of spores in the colonic tract which can be shedded to the environment for up to 1 to 4 weeks after CDI treatment [4]. Indeed, *in vitro* work has demonstrated that *C. difficile* spores adhere particularly well to intestinal epithelial cells in culture [5]. Dormant *C. difficile* spores are impermeable to all known antibiotic treatments [6], [7]. These persistent spores then germinate, colonize and produce recurrent CDI episodes [7]. *C. difficile* spores germinate in presence of cholates and its derivatives [8], [9] but germination is more efficient in the presence of certain amino acids that act as co-germinants [10]. The massive macrophage and neutrophil recruitment during the course of CDI suggests that there must be some sort of interaction between *C. difficile* and the innate immune system. Indeed, recent studies have demonstrated that Toll-like receptor 4 and the nucleotide-binding oligomerization domain 1 (Nod1) recognizes the *C. difficile* vegetative cells and mediate protection against CDI [11], [12].

Once phagocytosed, the fate of bacterial spores will vary depending on their specific virulence traits that will enable them to either escape of or to modulate the host innate immune system. For example, depending on the germination ability of *Clostridium perfringens* spores their fate is significantly different [13]. Isolates with germination proficient spores were efficiently inactivated during macrophage infection, while those that germinated poorly were able to survive for extended periods of time inside macrophages [13]. The main factors involved in resistance of *C. perfringens* spores are: i) the spore maturation proteins that regulate spore water content [14]; ii) α/β-type small acid soluble proteins (SASPs) that bind and saturate the spores’ DNA [15]–[17]; and iii) the SpoVA proteins, which are involved in uptake of dipicolinic acid (DPA) and reduction of the spore core water content [18]. However, *C. perfringens* spores deficient in either of these factors were able to survive similarly as wild-type spores during infection with macrophages, suggesting that other ultrastructural properties of *C. perfringens* spores are involved in macrophage-resistance [13]. Indeed, studies [19]–[21] demonstrated that *Bacillus anthracis* wild-type spores are efficiently phagocytosed and upon germination inside the phagolysosome they were efficiently killed by macrophages. However, *B. anthracis* germination deficient (Δ*gerH)* spores were able to survive longer periods of time than germination proficient wild-type spores, indicating that spore survival inside macrophages is dependent on the ability of spores to remain dormant [22].

The ability of *C. difficile* spores to germinate mainly in presence of the co-germinants taurocholate and glycine or other amino acids [8], [10], suggests that since at least cholates are not part of the phagolysosomes environment, the fate of *C. difficile* spores during macrophage infection might be significantly different than that reported for spores of *C. perfringens* and *B. anthracis*. Therefore, in this study, we evaluated the interactions of *C. difficile* spores with Raw 264.7 macrophages. By using electron microscopy, fluorescence microscopy and cell viability assays we show that *C. difficile* spores are efficiently recognized and phagocytosed by Raw 264.7 cells. However, *C. difficile* spores do not germinate inside macrophages and are able to survive, and produce cytotoxic effects to Raw 264.7 cells. These findings indicate that *C. difficile* spores survive attacks of phagocytic cells.

## Materials and Methods

### Bacterial Strains, Cell Lines, and Chemical Reagents

*C. difficile* strains 630 (*tcdA* ^+^, *tcdB* ^+^, *tcdC* ^+^, *ctdA* ^−^, and *ctdB* ^−^) [23] and Pitt177 (*tcdA* ^+^, *tcdB* ^+^, *tcdC* ^+^, *ctdA* ^+^, and *ctdB* ^+^) are described elsewhere [24], [25]. *C. difficile* strain Pitt177 was isolated from patients presenting clinical symptoms of CDI in a tertiary care hospital in Pittsburg, U.S.A. [25]. Raw 264.7 murine macrophages (ATCC, U.S.A) were routinely grown in Dulbecco’s modified Eagle’s minimum essential medium (DMEM) (Invitrogen) supplemented with 10% (V/V) fetal bovine serum (ATCC, Manassas) and incubated at 37°C in 5% CO_2_ humidified atmosphere. *B. subtilis* strain PS832 wild-type [26] was kindly provided by Dr. Peter Setlow.

### Spore Preparation, Purification and Sonication

*C. difficile* spores were prepared as previously described [8]. Briefly, Brain Heart Infusion broth (Difco) supplemented with 0.5% yeast extract (Difco) (BHIS) was inoculated with *C. difficile* and incubated overnight under anaerobic conditions at 37°C. Overnight cultures were diluted to an OD_600_ of 0.2, plated onto BHIS agar and incubated under anaerobic conditions at 37°C for 10 days [8]. BHIS agar plates were flooded with 10 ml of ice cold sterile distilled water, washed by repeated centrifugation and resuspension followed by purification through 50% HistoDenz and washed five times to eliminate traces of HistoDenz. Spore suspensions were >99% free of vegetative cells, sporulating cells and cell debris as determined by phase contrast microscopy. Spore suspensions were stored at −80°C until use. *B. sutbilis* spores were prepared by growing for ∼72 h at 37°C on BHI agar plates under aerobic conditions, and spores were purified as described [27].

To remove the *C. difficile* spore exosporium layer, *C. difficile* spores were resuspended in 50 mM Tris-HCl 0.5 mM EDTA buffer (pH 7.5) and sonicated with maximum power (20 Watts) for 10 1-min burst separated by 3 min of cooling on ice-cold water as previously described [5]. This sonication protocol has been previously shown by transmission electron microscopy to remove spore surface material affecting the *C. difficile* spore’s surface hydrophobicity by ∼60% and their ability to adhere to epithelial cells [5]. As a marker for the removal of the spores’ exosporium material we measured the change of the spore’s surface hydrophobicity, which was ∼60 and 25% in untreated and sonicated spores, respectively. These results were similar to those reported previously [5]. Sonicated *C. difficile* spores were stored at −20°C until use.

### Infection of Raw 264.7 Cells with *C. difficile* Spores

For electron microscopy experiments, Raw 264.7 cells (∼6×10^5^ cells/well) were seeded onto cover slips coated with poly-lysine (BD, USA) on 24-well culture plates for 24-h, rinsed 3 times with Dubelcco’s PBS (DPBS) (Lonza) and infected at a multiplicity of infection (MOI) of 10 for 30 min with 100 µl of DMEM containing *C. difficile* spores (∼6×10^6^ spores). Unbound spores were rinsed off with three washes of DPBS, and some samples of infected macrophages further incubated for 24 h at 37°C with 5% CO_2_. Samples were fixed with freshly prepared 2.5% glutaraldehyde-1% paraformaldehyde in 0.1 M cacodylate buffer (pH 7.2) for overnight at 4°C. Secondary fixation was performed with 1% osmium tetroxide-0.1 M cacodylate buffer (pH 7.2), rinsed in cacodelyte buffer and stained for 30 min with 1% tannic acid. Samples were dehydrated with a step-wise acetone gradient of 30% (stained with 2% uranyl acetate at this stage) for 30 min, 50% for 30 min, 70% for overnight, 90% for 30 min, and twice with 100% acetone. Dehydrated samples were embedded in spurs at a ratio of acetone:spurs of 3∶1, 1∶1 and 1∶3 for 40 min each and finally resuspended in spurs for 4 h and baked overnight at 65°C. Thin sections were obtained using a microtome and were placed on a glow discharge carbon-coated grids for negative staining and double lead stained with 2% uranyl acetate and lead citrate. Samples were analyzed at 80 kV with a Philips EM300 transmission electron microscopy (TEM) at the Electron Microscopy Facility at Oregon State University and with a Phillips Tecnai 12 Bio Twin at the Electron Microscopy facility at Pontificia Universdad Católica de Chile.

For scanning electron microscopy (SEM), Raw 264.7 cells were infected as described above and fixed with 2.5% glutaraldehyde-1% paraformaldehyde in 0.1 M cacodylate buffer, and serially dehydrated with acetone: 30% for 20 min, 50% for 20 min, 75% for 20 min, 90% for 20 min and twice with 100% for 20 min. Dehydrated samples were subjected to critical point drying and coated with gold and palladium and analyzed with a FEI Quanta 600 PEG at the electron microscopy facility of Oregon State University.

### Fluorescent Labeling of *C. difficile* Spores

Purified *C. difficile* spores were labeled with Alexa Fluor 488 protein labeling kit (Molecular Probes) according to the manufacture’s specification. Briefly, 1 ml of a spore suspension with a calculated OD_600_ 50 was resuspended in 1 ml of 0.1 M sodium bicarbonate (pH ∼8.3), mixed well with 200 µl of 0.1 M sodium bicarbonate-0.02 µg/ml Alexa Fluor 488 dye and incubated at room temperature for 30 min. Unconjugated dye was removed by centrifugation and the labeled spores were washed five times with sterile distilled water. Alexa Fluor 488-labeled spores were biotin labeled by resuspending the spore suspension in 0.1 M sodium bicarbonate buffer (pH 8.2)−0.3 mg/ml of Sulfo-NHS-LC-Biotin (Molecular Probes, Invitrogen, U.S.A.) and incubated for 45 min at room temperature. Biotin- Alexa Fluor 488-labeled *C. difficile* spores were washed, counted with a Heber Bacteria Counting ChamberZ30 (Hawksley, UK) and stored at −20°C until use. The fluorescent labeling did not decrease spore viability (data not shown).

### Visualization of Labeled Spores by Fluorescence Microscopy

To quantify adherence and phagocytosis of *C. difficile* spores by Raw 264.7 cells, macrophages were seeded at a concentration of 6×10^5^ cells/well onto 8 well culture slides (FD Falcon) and incubated at 37°C for 24 h. Raw 264.7 cells were washed twice with DPBS (Gibco), and incubated with 100 µl of DMEM containing biotin- and Alexa Fluor 488-labeled *C. difficile* spores at an MOI of 4 for 30 min at 37°C. Wells were rinsed twice with DPBS to remove any unbound spores, and fixed for 15 min at room temperature with 200 µl of freshly prepared 4% paraformaldehyde. Fixed macrophages were rinsed twice with DPBS and extracellular *C. difficile* spores were labeled at room temperature for 50 min with streptavidin-Alexa350 (Molecular Probes, Invitrogen, CA) diluted 1∶100 in DPBS-1% Bovine Serum Albumin (Sigma-Aldrich), and rinsed three times with DPBS. Cells were permeabilized with 0.06% Triton X-100 in DPBS for 15 min at room temperature, rinsed three times with DPBS, stained for F-actin with 1 U of Alexa Fluor 568-phalloidin (Molecular Probes) for 30 min and rinsed three times with DPBS. Samples were air dried, sealed with nail polish and analyzed in a DM4008B fluorescence microscope (Leica, Wetzier, Germany). Internalized spores were identified as green spores that were not labeled blue by streptavidin-Alexa350, while extracellular or adherent spores were identified as green spores that superimpose with the macrophage’s F-actin cytoskeleton and that were stained blue by streptavidin-Alexa350. For each test conditions, pictures were taken for at least ∼3000 spores in 100 fields, and photomicrographs were prepared with Adobe Photoshop and Microsoft Picture Manager Software and extracellular and internalized spores counted in by eye. All experiments were performed at least three times.

To evaluate if *C. difficile* spores were internalized through an actin polymerization dependent mechanism by Raw 264.7 cells, 10 µM of cytochalasin D was added to each well prior to the infection, and maintained for the duration of the experiment. Infected macrophages were washed and treated for fluorescence microscopy analysis as described above. To evaluate the effect of complement on binding and phagocytosis, *C. difficile* spores were incubated with DMEM-10% untreated or heat inactivated fetal bovine serum (FBS) (Gibco, U.S.A.) or with DMEM-10% heat inactivated FBS supplemented with 3–4 week rabbit complement (diluted 1∶50 in heat inactivated FBS) (Biotech Brand) for 30 min prior to infection. Infected cells were washed and treated for fluorescence microscopy as described above.

### Killing of *C. difficile* Spores by Raw 264.7 Cells

To quantify killing of *C. difficile* spores, macrophages were seeded 24 h prior to infection in 24-well plates. Macrophages were rinsed with DPBS and infected with 200 µl of DMEM containing *C. difficile* spores at various MOI’s. After 30 min of incubation at 37°C, infected macrophages were washed three times with DMEM, resuspended in 400 µl of DMEM in absence of FBS and incubated for various periods of time under aerobic conditions at 37°C in 5% CO_2_. Viability of *C. difficile* spores was determined at 0.5, 5, and 24 h after infection by lysing infected macrophages with 0.01% Triton X-100, serially diluting into DPBS and plated onto BHI agar plates (Difco) supplemented with 0.1% sodium taurocholate (ST) and incubated for 24 hrs anaerobically at 37°C for colony counts, no additional colonies were observed upon further incubation periods. In some experiments, *C. difficile* spores were pre-incubated with or without 1.0% ST, 0.1% ST-5 mM L-glycine (STG), 50% human serum (HS) in DMEM, or with 50% HS-STG for 30 min before infecting Raw 264.7 cells. Initial spore counts were quantified by plating serially diluted aliquots onto BHI agar plates.

To evaluate outgrowth of STG-treated *C. diffcile* spores during infection of macrophages, Raw 264.7 cells were infected for 30 min with STG-treated spores incubated for 24 h under either aerobic with 5%-CO_2_ or anaerobic conditions in a anaerobic chamber Bactron III-2 (OR, U.S.A.), fixed with paraformaldehyde as described above, stained with DAPI and examined by fluorescence microscopy.

### Killing of *C. difficile* Vegetative Cells by Raw 264.7 Cells

To quantify killing of *C. difficile* vegetative cells by Raw 264.7 cells, *C. difficile* cells were quantified in overnight cultures (∼18 h) with a Heber Bacteria Counting Chamber Z300 (Hawksley, UK) and used to infect at an MOI of 10 as described above. Infection under aerobic conditions was done with 5%-CO_2_, while for anaerobic conditions, Raw 264.7 cells were preincubated for 1 h in an anaerobic chamber (Bactron III-2, Shellab, OR, U.S.A.) in DMEM medium that had been previously reduced for 72 h under anaerobic conditions, and infected with *C. difficile* cells resuspended in prereduced DMEM medium. All manipulations requiring anaerobic conditions were done inside a Bactron III-2 anaerobic workstation (Shellab, OR, U.S.A). Initial cell counts were quantified by plating serially diluted aliquots onto BHI agar plates.

### Live/dead Assay of Raw 264.7 Cells

To evaluate the cytotoxic effects of *C. difficile* spores on Raw 264.7 cells during the course of infection, Raw 264.7 cells (∼5×10^5^) seeded in 96 well plates were infected with *C. difficile* spores at various MOI for 30 min. Raw 264.7 cells were washed twice with DPBS to remove unbound cells and infected monolayers of Raw 264.7 cells were incubated in DMEM in absence of FBS under aerobic conditions with 5% CO_2_ for 24 and 48 hrs. Cytotoxicity was evaluated using the LIVE/DEAD Viability/Cytotoxicity Kit for mammalian cells (Molecular Probes, OR, U.S.A.). Live cells are distinguished by the enzymatic production of intensively fluorescent calcein (Ex/Em 494/517 nm) from non-fluorescent cell-permeant calcein AM through intracellular esterease activity. Dead cells are detected with ethidium homodimer-1 (Ex/Em 528/617 nm) that enters cells with damaged membranes and binds to nucleic acids increasing 40-fold its fluorescence intensity, producing a bright red fluorescence in dead cells. In live cells, ethidium homodimer-1 is excluded by the intact plasma membrane. Inherently low levels of background fluorescence were detected in control treatments (data not shown). Dead Raw 264.7 cell control were Raw 264.7 cells treated with 0.06% Triton 100×10 min prior to addition of ethidium homodimer-1, while live Raw 264.7 cell control were monolayers of Raw 264.7 cells incubated for 24 and 48 h in absence of *C. difficile* spores. Fluorescence was measured in a Infinite 200 PRO (Tecan, U.S.A.) using appropriate filters for ethidium homodimer-1 (filter with excitation at 535 nm and emission at 620 nm) and calcein (filter with excitation at 485 nm and emission at 535 nm). The percentage of dead cells was calculated using the following formula: % Dead Cells = [[F(620 nm)_sample_–F(620 nm)_minimum_]/[F(620 nm)_maximum_–F(620 nm)_minimum_ ]]×100%; where F(620 nm) is fluorescence intensity at a wavelength of 620 nm, F(620 nm)_sample_ is fluorescence of Raw 264.7 cells infected with spores, F(620 nm)_minimum_ is fluorescence of Raw 264.7 cells with no spores, F(620 nm)_maximum_ is fluorescence of Raw 264.7 cells treated with 0.06% Triton 100X. The percentage of live cells was calculated using the following formula: % Live Cells = [[F(535 nm)_sample_–F(535 nm)_minimum_]/[F(535 nm)_maximum_–F(535 nm)_minimum_ ]]×100%; where F(535 nm) is fluorescence intensity at a wavelength of 535 nm, F(535 nm)_sample_ is fluorescence of Raw 264.7 cells infected with spores, F(535 nm)_minimum_ is fluorescence of Raw 264.7 cells treated with 0.06% Triton 100 X, F(535 nm)_maximum_ is fluorescence of Raw 264.7 cells with no spores. All experiment were done in triplicate and repeated at least three times.

### Statistical Analysis

All experiments were repeated at least three times. Error bars represent standard error from the mean. Statistical analysis in some experiments involved Student’s *t* test, used to identify significant difference between groups, or pairwise comparison with Least Significant Difference. Statistical difference was considered with a p<0.05. Statistical analysis was done with Statgraphics Centurion XVI (StatPoint Technologies, Inc).

## Results

### Characterization of Internalization of *C. difficile* Spores by Raw 264.7 Cells

**Figure 1:**
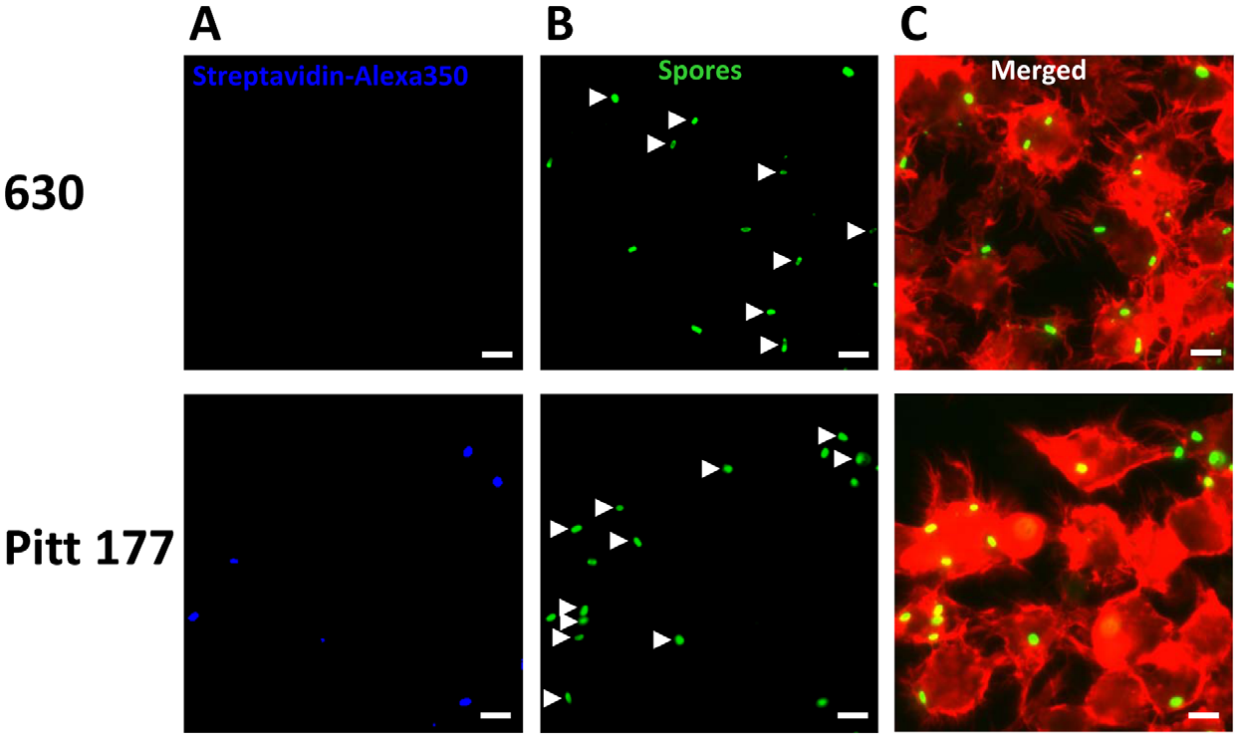
Representative fluorescence micrographs of internalization of *C. difficile* spores by Raw 264.7 cells. *C. difficile* spores were labeled with biotoin and Alexa Fluor 488 (green) prior to infection of monolayers of Raw 264.7 cells (red). Infected Raw 264.7 cells were washed; fixed and extracellular spores were stained with Streptavidin-Alexa Fluor 350 conjugate (blue), stained for F-actin and analyzed by fluorescence microscopy as described in Methods section. Representative micrographs of phagocytosis of *C. difficile* spores are shown: A) Extracellular *C. difficile* spores (blue); B) Total *C. difficile* spores (green); C) Merged images. Bars represent 5 µm. White arrows highlight internalized spores.

To investigate binding and phagocytosis of *C. difficile* spores by Raw 264.7 cells, *C. difficile* spores were labeled with Alexa Fluor 488 and biotin and then used to infect Raw 264.7 cells. The spore interaction to and spore internalization by Raw 264.7 cells was evaluated by fluorescence microscopy (Fig. 1), with green *C. difficile* spores stained blue corresponding to extracellular spores. Our preliminary results demonstrated that Alexa-488 and biotin labeling did not affect Raw 264.7 cells to recognize *C. difficile* spores, and Raw 264.7 cells were able to adhere and phagocytose *C. difficile* spores better than THP-1 and J937 macrophage-like cell lines (data not shown). Therefore, Raw 264.7 cells were used in all subsequent experiments.

**Figure 2:**
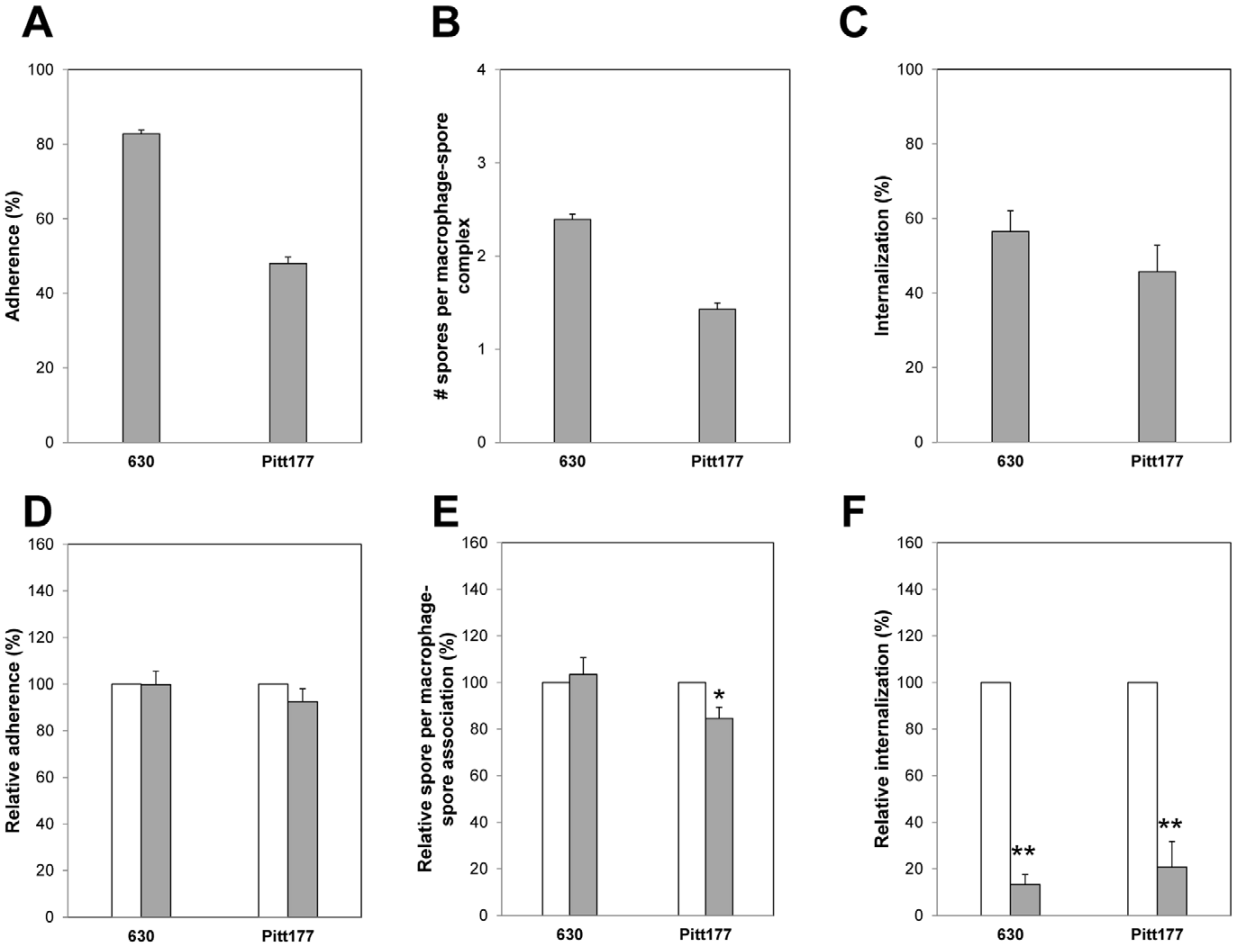
Adherence and internalization of *C. difficile* spores by Raw 264.7 cells. Monolayers of Raw 264.7 cells were infected at an MOI of 10 with Alexa- and biotoin-labeled *C. difficile* spores of strains 630 and Pitt177, unbound spores washed, and samples prepared for fluorescence microscopy. Percentage of raw macrophages with at least one spore (A), number of spores per macrophage complex (B), and percentage of intracellular spores (C) were quantified as described in Methods section. D, E, F) The effect of cytochalasin D on the relative binding of spores to Raw 264.7 cells (D), relative number of spores per Raw-spore complex (E), and relative percentage of internalization (F) was evaluated without (white bars) and with 1 µM cytochalasin D (grey bars). Relative values refer to the relative percentage of Raw 264.7 cells with at least one spore (D), relative number of spores per Raw-spore complex (E), and relative percentage of internalization (F) in presence of cytochalasin D normalized to the culture medium control. Results are combined from at least three independent experiments and error bars are standard error of the mean. Asterisks (*) denote statistical difference at p<0.05, and double asterisks (**) denote statistical difference at p<0.01 compared to culture medium control.

Infection of Raw 264.7 cells with *C. difficile* strain 630 spores in DMEM and absence of serum showed that 83% of macrophages attached to at least one spores (Fig. 2A), with each Raw 264.7 cell binding to an average of 2.5 spores (Fig. 2B). Interestingly, 59% of all *C. difficile* 630 spores attached to Raw 264.7 cells were efficiently internalized after 30 min of incubation (Fig. 2C). Incubation for up to 3 h did not increase adherence and internalization of *C. difficile* spores by Raw 264.7 cells (data not shown). When Raw 264.7 cells were infected with spores of *C. difficile* strain Pitt177, only 40% of Raw 264.7 cells (Fig. 2A) were attached to at least one spore, with an average ∼1.5 spores per Raw 264.7 cell (Fig. 2B), results that were significantly (p<0.01) lower than those observed with *C. difficile* strain 630 spores (Fig. 2A,B). Similar to *C. difficile* strain 630 spores, 50% of adhered *C. difficile* strain Pitt177 spores had been internalized by Raw 264.7 cells (Fig. 2C).

Interestingly, no effects of cytochalasin D, an actin polymerization inhibitor [28], on adherence of spores of *C. difficile* strain 630 to Raw 264.7 cell was observed (Fig. 2D, E). Although there was no significant decrease in the percentage of Raw 264.7 cells with at least one spore of *C. difficile* strain Pitt177 (Fig. 2D), there was a slight, but significant (p<0.05) decrease in the number of Pitt177 spores per macrophage-spore complex (Fig. 2E). However, a significant (p<0.001) decrease in internalization of spores by Raw 264.7 cell was observed (Fig. 2F), indicating that the internalization of *C. difficile* spores by Raw 264.7 cells is dependent on actin polymerization.

### Complement Decreases Internalization of C. difficile Spores by Raw 264.7 Cells

**Figure 3:**
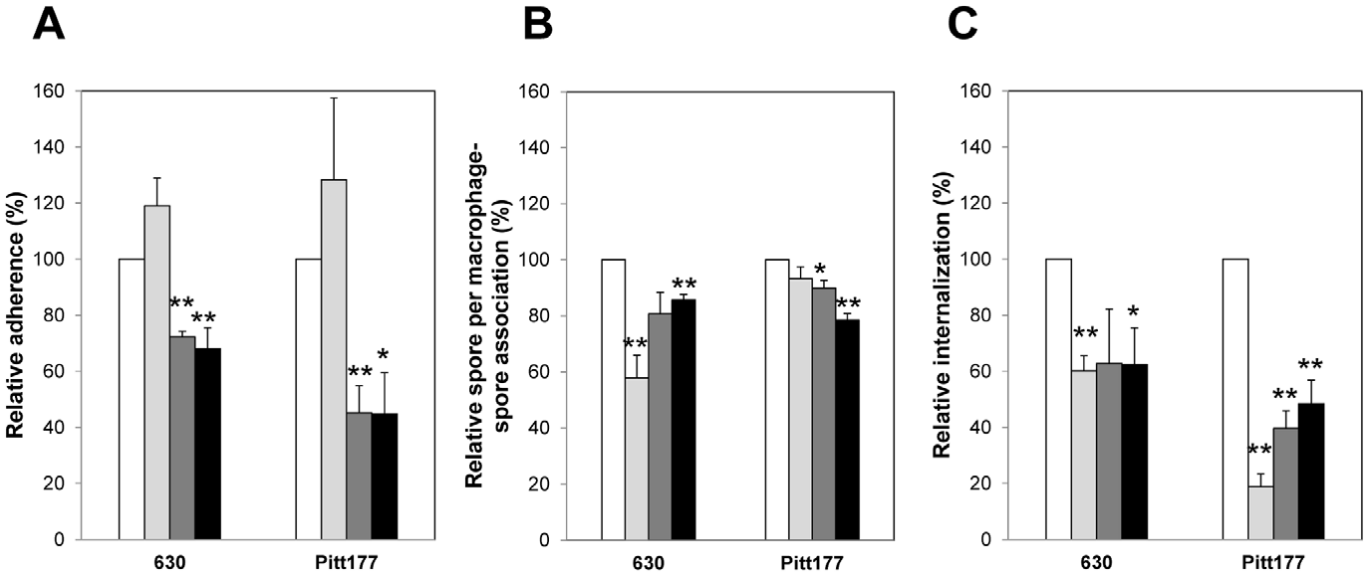
Complement decreases internalization of *C. difficile* spores by Raw 264.7 cells. Alexa- biotin-labeled *C. difficile* spores of strains 630 and Pitt177 were incubated for 30 min with culture medium (white bars), fetal bovine serum (FBS) (light grey bars), heat inactivated FBS (dark grey bars), and heat inactivated FBS supplemented with rabbit complement (black bars) prior to infection of monolayers of Raw 264.7 cells as described figure legend of Figure 2. The relative percentage of Raw 264.7 cells with at least one spore (A), relative number of spores per Raw-spore complex (B), and relative percentage of internalization (C) were quantified and calculated as described in Methods section and legend of Figure 2. Results are combined from at least three independent experiments and error bars are standard error of the mean. Asterisks (*) denote statistical difference at p<0.05, and double asterisks (**) denote statistical difference at p<0.01 compared to culture medium control.

Complement proteins are constitutively present in the serum and can opsonize bacteria nonspecifically contributing to pathogen clearance [29]. Therefore, we evaluated if complement might play a role in recognition and phagocytosis of *C. difficile* spores by Raw 264.7 cells. Surprisingly, although the adherence of *C. difficile* spores to Raw 264.7 cells was not significantly affected by the presence of fetal bovine serum (FBS) than in absence of FBS (Fig. 3A), it was significantly (p<0.05) less in presence of heat-inactivated FBS (absence of complement) than in absence of FBS (Fig. 3A). The number of average spores per Raw 264.7 cell in presence of FBS significantly (p 0.01) decreased for spores of *C. difficile* strain 630 but not for spores of *C. difficile* strain Pitt177 (Fig. 3B). Heat-inactivated FBS produced a slight decrease in the number of average spores per Raw 264.7 cells for both *C. difficile* strains 630 and Pitt177 than that in DMEM alone (Fig. 3B). It was also striking to note that the presence of FBS or heat inactivated FBS also decreased the percentage of spore internalization by Raw 264.7 cells (Fig. 3C). Addition of complement did not restore the adherence and internalization of spores of both *C. difficile* strains 630 and Pitt177 by Raw 264.7 cells (Fig. 3A,B,C), indicating that Raw 264.7 cells are less able to recognize and internalize *C. difficile* spores through complement-mediated phagocytosis.

### Sonication Effects on Adherence and Internalization of *C. difficile* Spores by Raw 264.7 Cells

**Figure 4:**
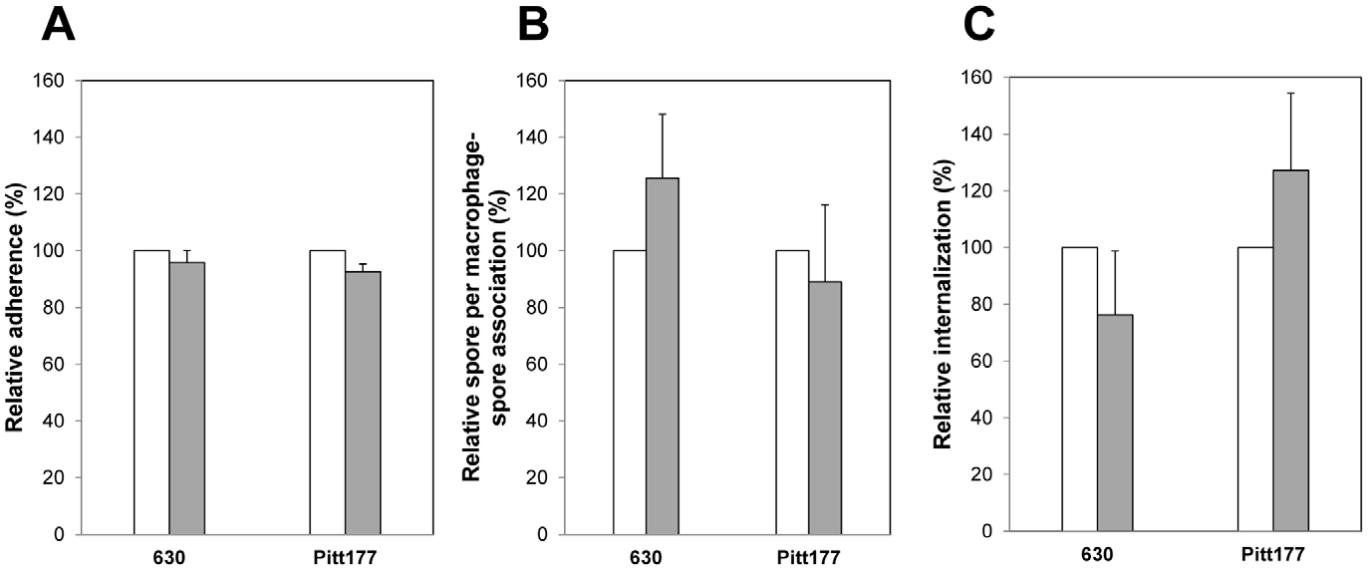
Sonication does not affect binding and internalization of *C. difficile* spores by Raw 264.7 cells. Monolayers of Raw 264.7 cells were infected with untreated (white bars) and sonicated (grey bars) *C. difficile* spores of strains 630 and Pitt177 at an MOI of 10 for 30 min, and analyzed by fluorescence microscopy for: relative percentage of Raw 264.7 cells with at least one spore (A), relative number of spores per Raw-spore complex (B), and relative percentage of internalization (C) as described in Methods section and in the legend of figure 2. Results are the average of at least three independent experiments and error bars are standard error of the mean.

Several species, such as *B. anthracis* and *B. cereus*, have a balloon-like structure that overlays the coats, called the exosporium [30]. Indeed, *C. difficile* seems to have an exosporium-like structure [5], [31]. Therefore, to evaluate if this structure might have motifs recognized by Raw 264.7 cells, *C. difficile* spores were sonicated, treatment that removes at least some of the exosporium material [5], and tested for adherence. There was no significant decrease in adherence of sonicated spores of *C. difficile* strains 630 and Pitt177 to Raw 264.7 cells (Fig. 4A). The number of spores that adhered to Raw 264.7 cells was not affected by sonication (Fig. 4B). Furthermore, there was no significant decrease in internalization of spores of *C. difficile* strains 630 and Pitt177 by Raw 264.7 cells (Fig. 4C). These results suggest that the motifs recognized by Raw 264.7 cells to bind and internalize *C. difficile* spores are not localized in the spores’ outermost layer that is affected by sonication; or alternatively, and most likely, the remaining exosporium structure contained sufficient conserved motifs recognized by Raw 264.7 cells.

### Raw 264.7 Cells Efficiently Recognize and Phagocytose *C. difficile* Spores

**Figure 5:**
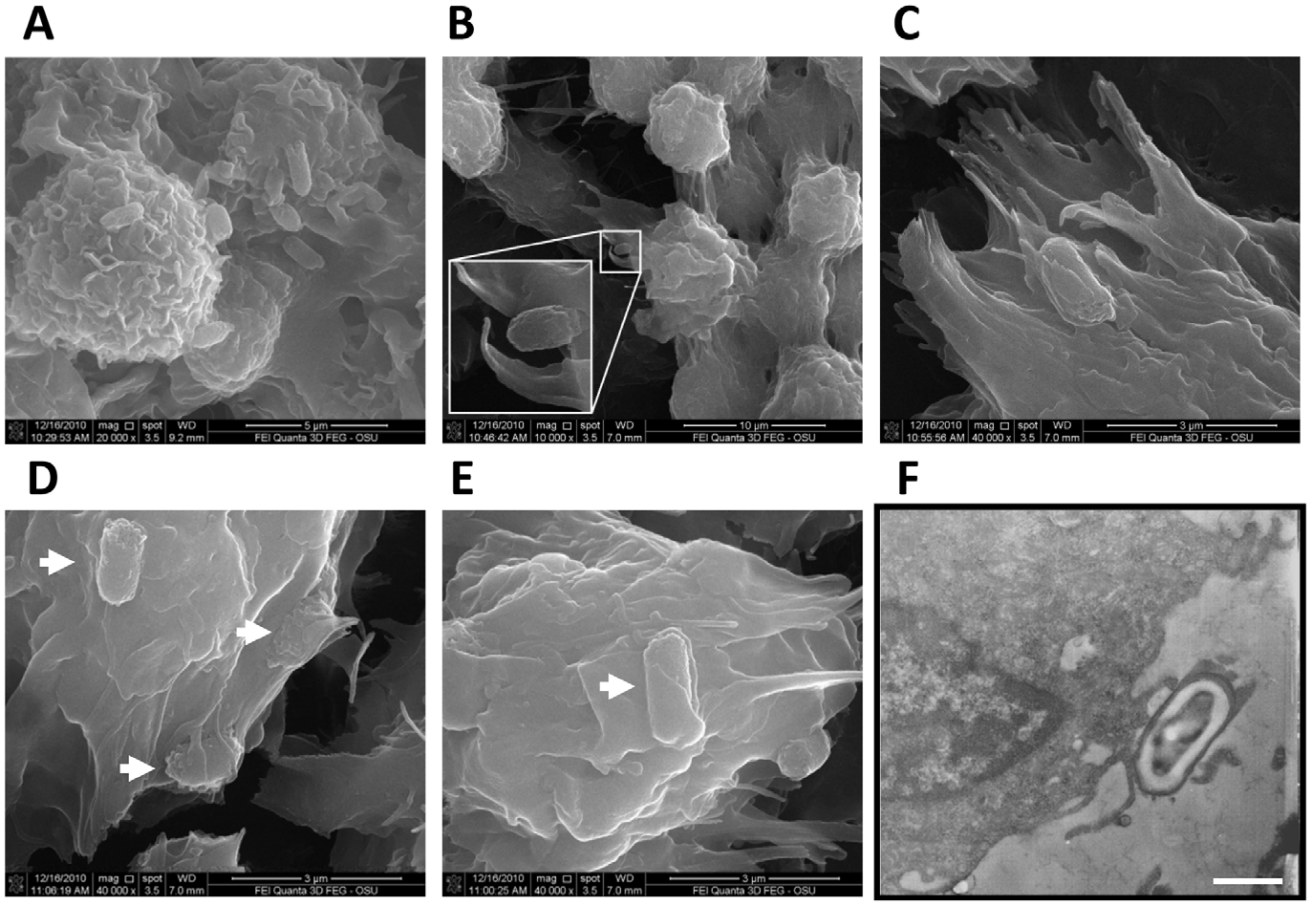
Raw 264.7 cells bind and phagocytose *C. difficile* spores. A,B,C,D,E,F) SEM of Raw 264.7 cells infected with *C. difficile* spores under aerobic conditions. Note the active phagocytosis of the spores by Raw 264.7 cells in both panels. White arrows denote coiling phagocytosis. F) Transmission electron microscopy (TEM) of Raw 264.7 cells infected with *C. difficile* 630 spores at an MOI of 10 for 30 min under aerobic conditions. The area of adherence of *C. difficile* spores occurred at patchy regions at the end of protrusions from the surface of Raw 264.7 cells. Bar: 1 µm.

Scanning electron micrographs (SEM) confirmed our above observations that Raw 264.7 cells can bind more than one *C. difficile* spores (Fig. 5A). During the first 30 min of infection, Raw 264.7 cells actively phagocytosed spores, and this seems to be associated in part by an actin-dependent mechanism that produces membrane ruffling (Fig. 5B, C). In addition, some spores are being entrapped by Raw 264.7 cells’ membrane through a mechanism similar to coiling phagocytosis (Fig. 5D,E) [32]. Transmission electron microscopy (TEM) of spore-infected Raw 264.7 cells showed that the site of adherence of *C. difficile* spores to Raw 264.7 cells can also occur at the end of protrusions from the surface of Raw 264.7 cytoplasmic membrane (Fig. 5F). Collectively, these results suggest that phagocytosis of *C. difficile* spores by Raw 264.7 cells might be mediated through various phagocytic pathways [33].

### *C. difficile* Spores Survive Inside Raw 264.7 Cells

**Figure 6:**
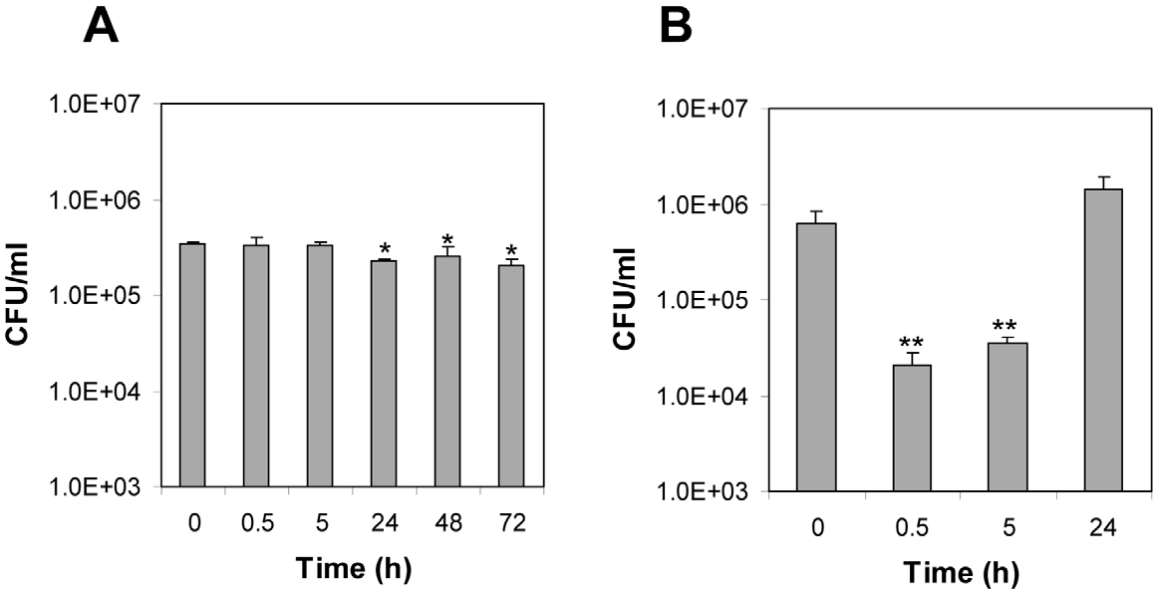
Survival of *C. difficile* spores and vegetative cells during infection of Raw 264.7 macrophages. Monolayers of Raw 264.7 cells were infected at an MOI of 10 with *C. difficile* strain 630: A) spores; B) vegetative cells, and unbound spores and vegetative cells rinsed off and further incubated under aerobic conditions for various periods of time and spore or vegetative cell viability was determined as described in Methods section. Results are the average of at least three independent experiments and error bars are standard error of the mean. Asterisks (*) denote statistical difference at p<0.05, and double asterisks (**) denote statistical difference at p<0.01 compared to time 0 h.

Since *C. difficile* spores were efficiently recognized and internalized by Raw 264.7 cells, we evaluated if these spores could survive inside Raw 264.7 cells under aerobic conditions. Results demonstrate that no significant reduction in spore viability, measured by spores’ colony forming ability, was observed after 5 h of infection with Raw 264.7 cells at an MOI of 10 (Fig. 6A). Extension of infection periods to 24 h showed a slight but significant (p<0.05) reduction in colony forming ability (Fig. 6A). Strikingly, extending the length of infection up to 48–72 h at an MOI of 10 did not produce a further decrease in spore viability (Fig. 6A). Similar results were observed when Raw 264.7 cells were infected with *C. difficile* spores at an MOI of 1 (data not shown). These results suggest that *C. difficile* spores are well suited to survive inside phagocytic cells.

Next, we evaluated the survival of *C. difficile* vegetative cells during macrophage infection for comparative purposes. More than three decimal reductions of vegetative cells of *C. difficile* strain 630 were observed during the first 30 min of infection with Raw 264.7 cells under aerobic conditions and no viable counts were detected after 5 h of infection. This killing was due to presence of oxygen rather than Raw 264.7 cells, since in absence of Raw 264.7 cells similar degree of killing of vegetative cells of *C. difficile* strain 630 was observed (data not shown). Since *C. difficile* is a strictly anaerobic bacterium that lacks most defense mechanisms against reactive oxygen species [34], infection experiments were repeated under anaerobic conditions. Macrophages have been previously reported to be able to survive up to 48 h under anaerobic conditions [35]. Prior to infection, Raw 264.7 cells and DMEM medium were prereduced for 1 and 72 h, respectively. After 24 h of incubation under anaerobic condition, more than 95% of Raw 264.7 cells remained viable as determined by trypan blue viability assay (data not shown). Interestingly, a significant (p<0.01) decrease on viability of *C. difficile* strain 630 vegetative cells was observed after 30 min of infection (Fig. 6B). After 5 h of incubation under anaerobic conditions, there was a slight increase in viable cell counts (Fig. 6B). Extending the infection time to 24 h demonstrated that *C. difficile* cells were able to out-grow Raw 264.7 cells (Fig. 6B). These results suggest that: i) under aerobic conditions, *C. difficile* cells are easily killed by Raw 264.7 cells; ii) under anaerobic conditions, although there is significant killing of *C. difficile* cells, the surviving cells are able to grow.

### *C. difficile* Spores Remain Intact Inside the Phagosome of Raw 264.7 Cells

**Figure 7:**
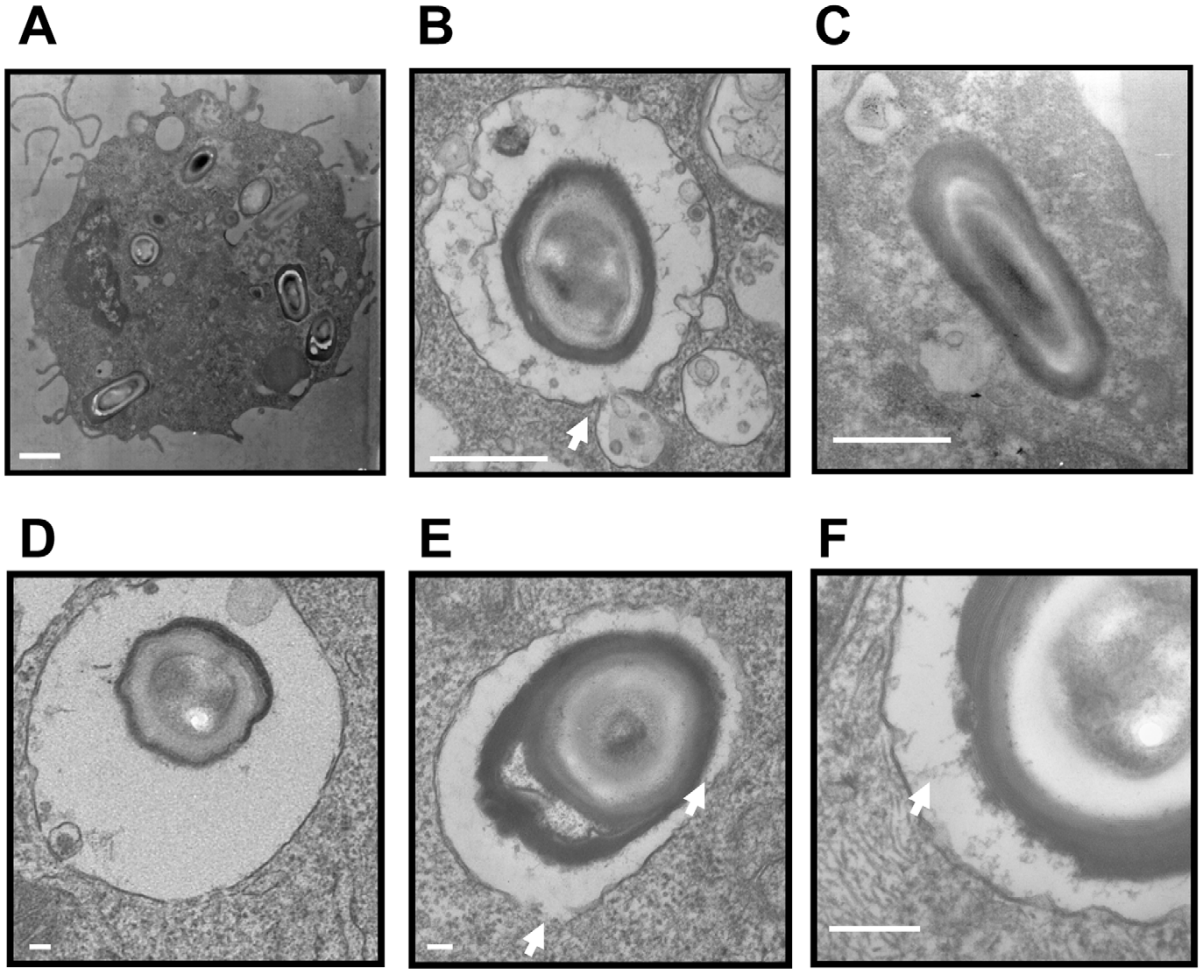
*C. difficile* spores remain intact inside the phagosome of Raw 264.7 cells. TEM images of Raw 264.7 cells infected with *C. difficile* spores under aerobic conditions (A–C, E, F) and with *B. subtilis* spores (D). TEM micrographs were taken after 30 min (A,B and D–F) and 24 h of infection. A) TEM shows that *C. difficile* spores are efficiently phagosytosed by Raw 264.7 cells. B), Phagosome containing *C. difficile* spores fuses with lysosomes, white arrows denotes fusion of lysosomes with the phagosome. C), *C. difficile* spores remain intact after 24 h of infection with Raw 264.7 macrophages. D) TEM micrograph of phagosytosed *B. subtilis* spores by Raw 264.7 cells. Phagosome’s membrane remains intact. E) TEM image showing phagocytosed *C. difficile* spore in a phagosome with membrane damage, white arrows denote disrupted phagosome membrane. F) TEM image rendering direct interactions between the surface of *C. difficile* spores and the phagosome’s membrane. White scale bar is 500 nm for panels A–C, and 100 m, for panels D-F.

To understand better the fate of *C. difficile* spores once inside the macrophage, TEM images were obtained after infection of Raw 264.7 cells with *C. difficile* spores. Raw 264.7 cells were able to efficiently phagocytose several *C. difficile* strain 630 spores and keep them inside the phagosomes (Fig. 7A). Furthermore, lysosomes fused to the phagosomes containing *C. difficile* spores (Fig. 7B), suggesting that mature phagolysosome were formed and that the spores were attacked by the antimicrobial machinery of Raw 264.7 cells. Although it seems as if some surface layers might be detaching from *C. difficile* spores (Fig. 7B), it was most striking that the ultrastructure of spores remained intact even after 24 h of infection with Raw 264.7 cells (Fig. 7C), indicating that *C. difficile* spores were able to survive inside macrophages during infection. Most notably, from a total of 30 spores analyzed after 0.5 and 24 h of infection with Raw 264.7 cells, no germinated spore was detected (data not shown), suggesting that *C. difficile* spores remained dormant inside macrophages.

Since *C. difficile* strain 630 spores are able to survive inside the macrophage’s phagolysosome, we hypothesized that these spores might be modulating the phagolysosome’s activity through direct interaction with spore’s surface proteins. Indeed, once phagocytosed, the fate of pathogenic bacteria varies depending on their ability to escape the phagosome. For example, *B. anthracis* spores localize to the late phagolysosome [21], whereas *C. perfringens* vegetative cells escape from the phagosome by degrading the phagosome membrane [35]. Therefore, to gain more insight into the fate of *C. diffcile* spores during macrophages infection, we analyzed TEM images of phagosytosed *C. difficile* strain 630 spores after 30 min of infection. As a control, we infected Raw 264.7 cells with *B. subtilis* PS832 wild-type spores, and observed that *B. subtilis* spores remained inside the phagosome with no disruption of the phagosome’s membrane (Fig. 7D). In contrast, in Raw 264.7 cells infected with *C. difficile* spores, several phagosomes containing *C. difficile* spores had a disrupted membrane (Fig. 7E and data not shown). A closer examination revealed that the surface of *C. difficile* spores is closely interacting with the phagosome’s membrane (Fig. 7F). Collectively, these results clearly indicate that at least some *C. difficile* spore-containing phagosome loss their membrane integrity and that *C. difficile* spore surface interacts with the phagosome’s membrane.

### Factors Affecting the Survival of *C. difficile* Spores During Infection of Raw 264.7 Cells

**Figure 8:**
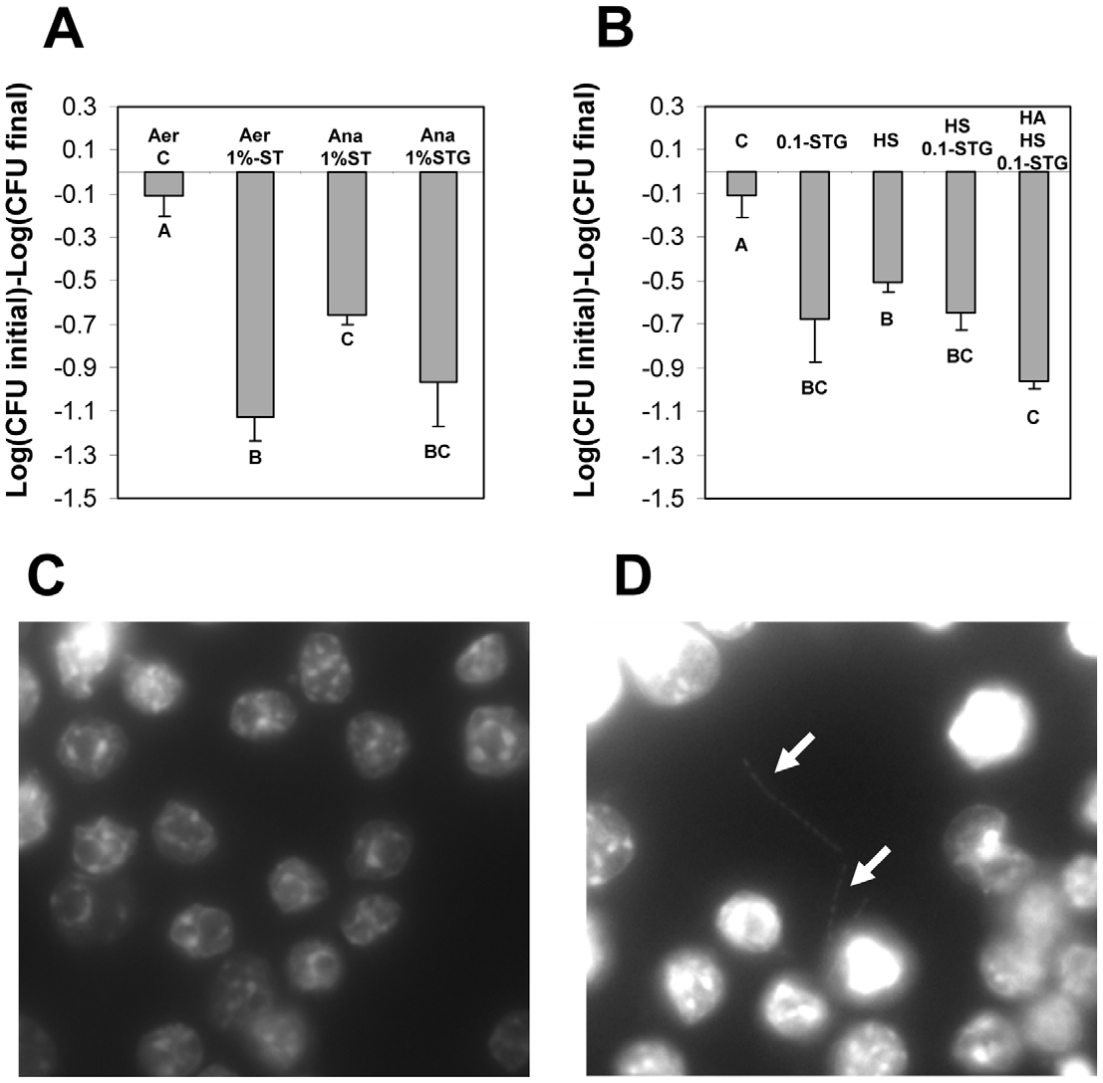
Factors affecting survival of *C. difficile* spores inside Raw 264.7 cells. A) Monolayers of Raw 264.7 cells were infected at an MOI of 10 with dormant *C. difficile* spores; and with spores incubated for 30 min with 1% sodium taurocholate (ST) in DMEM and 1% sodium taurocholate-5 mM L-glycine (STG) in DMEM. After 30 min of infection, unbound spores were washed, and Raw 264.7 cells were incubated in DMEM with no FBS under: Aer, aerobic conditions-5% CO_2_ for 24 h; Ana, anaerobic conditions for 24 h. Loss of spore viability was determined as described in Methods section. B) Monolayers of Raw 264.7 cells were infected with non-heat activated *C. difficile* spores treated for 30 min with: C, DMEM; 0.1-STG, 0.1% sodium taurocholate-5 mM L-glycine in DMEM; HS, 50% human serum in DMEM; HS 0.1-STG, 0.1% sodium taurocholate-5 mM L-glycine-50% human serum in DMEM; HA HS 0.1-STG, with heat activated *C. difficile* spores treated for 30 min with 0.1% sodium taurocholate-5 mM L-glycine-50% human serum in DMEM. Infected monolayers of Raw 264.7 cells were incubated for 24 h and viable spores were determined by plating aliquots of lysed Raw 264.7 cells onto BHIS agar plates as described in Methods section. Treatment C corresponds to data from Fig. 7A and is shown for comparative purposes. C,D) Fluorescence micrographs of Raw 264.7 cells infected with 1.0%-STG *C. difficile* spores. Infected Raw 264.7 cells were incubated for 24 h under aerobic (panel C) and anaerobic (panel D) conditions, fixed and DNA material of either Raw 264.7 cells and *C. difficile* vegetative cells were stained with DAPI as described in Methods section. White arrows denote growing *C. difficile* vegetative cells. Results are the average of at least three independent experiments and error bars are standard error of the mean.

The ability of bacterial spores to survive inside macrophages depends on their germination capabilities inside the macrophage environment [13], [22]. In addition, host factors with muramidase activity have been reported to trigger germination of *C. perfringens* and *B. anthracis* spores lacking the PG cortex hydrolysis machinery [24], [36], [37]. In this context, we evaluated the survival of spores after infecting Raw 264.7 cells with germinant- and/or human serum- treated *C. difficile* spores. Initial experiments demonstrated that when Raw 264.7 cells were incubated with DMEM in absence of FBS for 24 h under either aerobic or anaerobic conditions, there was no decrease in viability of Raw 264.7 cells as determined by trypan blue viability assay (data not shown). As expected, during infection of Raw 264.7 cells with untreated *C. difficile* spores, no significant inactivation of spores was observed (Fig. 8A). However, when Raw 264.7 cells were infected with ST treated spores, a significant (p<0.05) increase in spore killing was observed under aerobic and anaerobic conditions (Fig. 8A), indicating that germinated spores are easily killed by Raw 264.7 cells. The amount of spore killing in anaerobic condition was lower than under aerobic conditions but higher than in absence of ST (Fig. 8A), supporting the fact that the killing of ST-treated spores is due to Raw 264.7 cells and that absence of oxygen might allow some extracellular germinated *C. difficile* spores to survive during macrophage infection and increase number of viable cells. Addition of the co-germinant L-glycine increased the amount of spore killing during infection of Raw 264.7 cells under anaerobic conditions (Fig. 8A). Fluorescence microscopy of infected Raw 264.7 cells with ST-treated *C. difficile* spores demonstrated significant spore outgrowth only during anaerobic incubation (Fig. 8C,D), supporting the fact that at least some ST-treated *C. difficile* spores are able to outgrow during macrophage infection under anaerobic conditions. Collectively, these results suggest that *C. difficile* spores treated with ST are easily killed by Raw 264.7 cells.

Next, experiments were carried out with reduced concentration of ST (0.1%) in presence of 5 mM of L-glycine (STG) to unmask the effect of other potential factors such as human serum (HS). Significant killing was observed when Raw 264.7 cells were infected with STG treated spores (Fig. 8B). Strikingly, when *C. difficile* spores were pre-incubated with HS prior to infection of Raw 264.7 cells, there was also a significant (p<0.05) increase in spore killing (Fig. 8B). Pretreatment of spores with HS and STG produced a slight but not significant increase in spore killing by Raw 264.7 cells (Fig. 8B). Heat treatment activates bacterial spore’s germinant receptors to initiate the nonreversible germination process [38]. Therefore, experiments were repeated with heat-activated spores incubated with HS and STG prior infection. There was a slight difference (p = 0.06) in the amount of spore killing between heat-activated versus non heat activated spores incubated with STG and HS. However, this difference became significant (p<0.01) when killing of heat-activated spores incubated with STG and HS was compared to that of non-heat activated spores incubated with HS (Fig. 8B). These results indicate that germination factors sensitize *C. difficile* spores to macrophage mediated killing.

### *C. difficile* Spores are Cytotoxic to Raw 264.7 Cells

**Figure 9:**
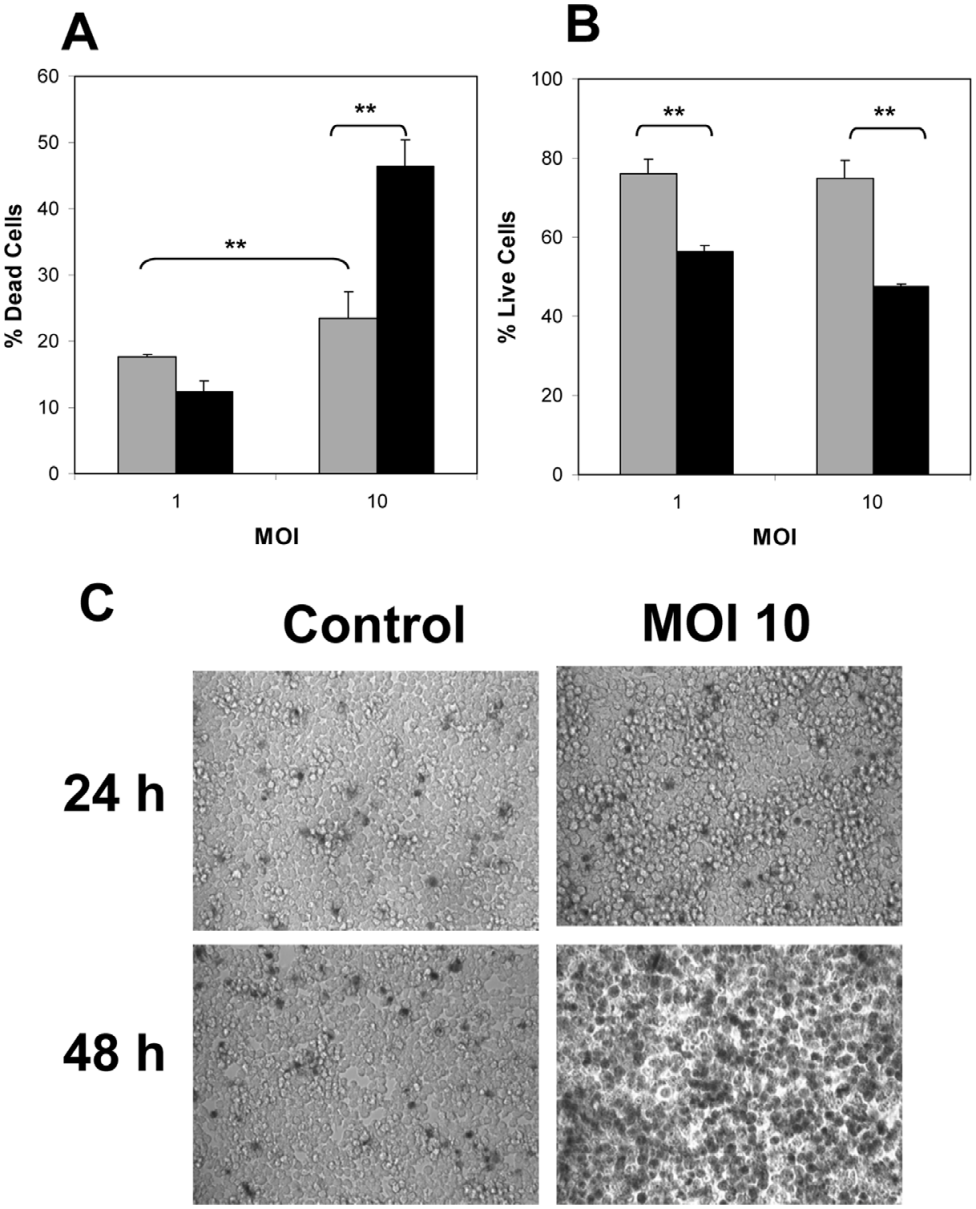
*C. difficile* spores affect viability of Raw 264.7 cells. Monolayers of Raw 264.7 cells were infected for 24 (grey bars) and 48 h (black bars) with *C. difficile* spores at MOIs of 1 and 10 and effects on Raw 264.7 cells was measured by quantification of: A) dead Raw 264.7 cells with ethidium homodimer-1; or B) live Raw 264.7 cells with calcein AM. Results were also confirmed with: C) cell viability assay with the membrane impermeable trypan blue as described in Methods section. Dark cells indicate disruption of the plasma membrane. Double asterisk indicate statistical significant difference (p<0.05) between treatments highlighted by brackets. Results are the average of at least three independent experiments and error bars are standard error of the mean.

Since above results suggest that *C. difficile* spores are able to survive and also interact with the phagosome’s membrane, we hypothesized that *C. difficile* spores might be cytotoxic to Raw 264.7 cells. When monolayers of Raw 264.7 cells were infected with *C. difficile* spores at an MOI of 1, some macrophage cell death was observed after 24 h of infection; however, cell death did not increase after 48 h of infection. 24 h of infection of Raw 264.7 cells with *C. difficile* spores at an MOI of 10 produced ∼50% of Raw 264.7 cell death (Fig. 9A). Also a significant loss of Raw 264.7 cell’s membrane integrity was observed (Fig. 9A) and this loss continued to increase until 48 h of incubation (Fig. 9A). Interestingly, when Raw 264.7 cells were infected with *C. difficile* spores at an MOI of 1 or 10, similar levels of reduction in Raw 264.7 cell viability were observed as measured by esterase activity. For example, after 24 h of infection, viability of Raw 264.7 cells decreased only by ∼30% and after 48 h by ∼50% (Fig. 9B). When results were confirmed by trypan blue exclusion assay, we observed that in concordance with above results, the majority of uninfected Raw 264.7 cells remained viable even after 48 h of incubation, however, significant cell death was observed on monolayers of Raw 264.7 cells infected with *C. difficile* spores at an MOI of 10 (Fig. 9C). Similar results were observed at an MOI of 1 (data not shown). Raw 264.7 cells alone incubated for up to 48 h had high levels of calcein fluorescence, and minimal levels of fluorescence due to ethidium homodimer-1 (data not shown) meaning that Raw 264.7 cells remained viable in absence of FBS. Collectively, these results indicate that *C. difficile* spores not only survive inside macrophages, but are also cytotoxic to Raw 264.7 cells over extended periods of infection.

## Discussion

Successful clearance of bacterial infections requires that the host’s immune system be able to recognize, phagocytose and kill pathogenic cells in host tissues. Although *C. difficile* is an enteric pathogen, during the course of CDI the enterotoxin TcdA and the cytotoxin TcdB not only induce epithelium damage but also the expression of numerous pro-immflamatory cytokines and chemokines, producing a massive recruitment of macrophages and neutrophils and the formation of pseudo-membranes [2]. Even though CDI is an enteric infection, and rarely systemic, macrophages do play a major role in host defense. Several studies have shown that the TLR4 and the Nod1 phagocytic receptors are essential for protection of CDI in mouse [11], [12], indicating that *C. difficile* cells and spores encounter phagocytic cells in the colonic environment. In this context, our current study provides numerous findings on the early stages of interactions between *C. difficile* spores and Raw 264.7 cells.

While the nature of phagocytic receptors [39] mediating *C. difficile* spore recognition by Raw 264.7 cells was not addressed in this work, we did observe that in absence of serum *C. difficile* spores were efficiently recognized by Raw 264.7 cells. It was most striking that more than half of *C. difficile* spores that adhered to Raw 264.7 cells were internalized. Our scanning electron micrographs suggest the uptake of *C. difficile* spores by Raw 264.7 cells might follow various phagocytic strategies which include membrane ruffling and coiling phagocytosis [32]. Although further studies will be required to fully characterize these pathways, they provide evidence that *C. difficile* spores are actively internalized by remodeling of the macrophages surface membrane through an actin mediated process [33], [39]. The significant reduction in internalization of *C. difficile* spores by Raw 264.7 cells in the presence of the actin polymerization inhibitor, cytochalasin D, supports that internalization is an actin-polymerization dependent process. It is worth noting that even in presence of cytochalasin D, ∼6–7% of adhered *C. difficile* spores were internalized, indicating that a small fraction of *C. difficile* spores enter Raw 264.7 cells through a phagocytosis-independent pathway. To the best of our knowledge, there are no reports of internalization of bacterial spores into phagocytic cells through a phagocytosis-independent pathway, thus the precise nature of this actin-polymerization independent internalization pathway is unknown and clearly requires further research.

Sonication treatment of *C. difficile* spores, which removes the majority (>60%) of the outermost exosporium-like structure (this study) including two ∼40-kDa protein species reported to have a role in adherence [5], did not affect the ability of Raw 264.7 cells to recognize and phagocytose *C. difficile* spores. This is presumably because remnants of the exosporium-like structure hold sufficient motifs recognized by Raw 264.7 cells to phagocytose *C. difficile* spores. Therefore, in an *in vivo* situation in the absence of some exosporium material, activated primary macrophages localized in the lamina propria, might efficiently recognize and phagocytose *C. difficile* spores. In addition, it was quite surprising to note that the percentage of *C. difficile* spore adherence of and phagocytosis by Raw 264.7 cells were lower for a clinical isolate Pitt177 than for the domesticated strain 630, suggesting that some sort of antigenic variation might have occurred on the surface of Pitt177 spores allowing it to evade attacks from the innate immune system. Unfortunately, our knowledge of the spore surface proteins is limited, and further studies on the identification of spore surface antigens will help uncover the reason for these differences.

Complement also plays a role in recognition and opsonization of bacterial pathogens [40]. Our current results showed that in presence of untreated or heat inactivated fetal bovine serum supplemented with complement there was a significant decrease of spore phagocytosis, suggesting that *C. difficile* spores might be able to modulate components of complement to evade the innate immune system. This phenomenon has been previously reported in numerous bacterial pathogens including *B. anthracis* [41]–[45]. Notably, *B. antharcis* spores’ surface α-enolase and Elongation factor Tu can bind plasminogen which in turn degrades deposited C3b resulting in a decrease in phagocytosis [41]. Although not yet proven, this raises the possibility that a similar mechanism might be applicable for *C. difficile* spores’ phagocytosis.

Once phagocytosed, survival of pathogenic bacteria is dependent on their ability to survive or escape the phagosome and/or inhibit the maturation of phagolysosome. Our results clearly suggest that *C. difficile* spores are unable to germinate during macrophage infection in absence of germinants. It was most interesting to note that *C. difficile* spores are well suited to survive inside Raw 264.7 murine-like macrophages for up to 72 h under aerobic conditions, which is due in part to their ability to remain dormant. It can be argued that survival of *C. difficile* spores during infection of Raw 264.7 cells could be due to macrophage cell death. While this was observed at an MOI of 10, where 49% of Raw 264.7 cells were dead after 48 h of infection, however, at an MOI of 1, only 12% of Raw 264.7 cells were dead, indicating that *C. difficile* spores’ survival ability is due to their intrinsic properties. In fact, no alterations in the spore ultrastructure was observed after 24 h of infection, indicating that *C. difficile* spores are able to resist successive attacks from the phagolysosome’s antimicrobial machinery. The ability of *C. difficile* spores to survive inside the macrophage environment is most likely to the lack of germination, since the macrophage environment lacks the co-germinants ST and L-glycine. Indeed, a recent study demonstrated that *B. anthracis* spores with mutations in the main *gerA* receptors were able to survive inside Raw 264.7 macrophages primarily due to their ability to remain dormant during infection of Raw 264.7 cells [46]. Germination-dependent survival has also been observed in *C. perfringens*, with spores of isolates that are able to germinate during macrophage infection being more easily killed by macrophages than with spores of isolates that are less able to germinate during infection [13]. Similar spore killing became evident when *C. difficile* spores were pre-incubated with STG or HS prior to infecting Raw 264.7 cells: HS increased the killing of *C. difficile* spores by Raw 264.7 cells. Serum lysozyme has been previously shown to trigger germination of *C. perfringens* spores through spore’s PG cortex hydrolysis [36], suggesting that similarly, HS lysozyme might be sensitizing *C. difficile* spores to macrophage killing, most likely by degrading the spore’s PG cortex. Since the environment inside the phagosome is cholate free, untreated *C. difficile* spores remain dormant and therefore are likely to persist even after macrophage death. It is worth noting that *C. difficile* vegetative cells were efficiently inactivated by Raw 264.7 cells under aerobic conditions, environment typically found in the sub-mucosal intestinal layers. However, although under anaerobic conditions *C. difficile* vegetative cells were first efficiently killed by a fixed number of Raw 264.7 cells, *C. difficile* cell counts increased over 24 h infection. It can be speculated that this might be the case in the mucosal environment and towards the colonic lumen; however, the constant infiltration of macrophages might produce a more host favorable outcome than the *in vitro* results shown in this work. Regardless of the fate of *C. difficile* vegetative cells, *C. difficile* spores were particularly resistant to macrophage killing under aerobic conditions. Collectively, these results provide support to the hypothesis that *C. difficile* spore survival inside macrophages is primarily due to their ability to remain dormant. The extent of inactivation of *C. difficile* spores by Raw 264.7 cells after 72 h of incubation (i.e., ∼0.6 decimal reduction), from a host perspective, is not effective in terms of pathogen-clearance by the innate immune system, and in the case of CDI, it might hold implications in persistence and high relapse episodes of CDI. Indeed, it might be possible that *C. difficile* isolates with high sproulation efficiencies persist in the host’s colonic tract due to the virtue of the *C. difficile* spore’s elevated resistance to the hosts’ immune system. Recent developments of animal models suited to study the role of *C. difficile* spores in the initiation and persistence of CDI [47] will facilitate our future investigation in this direction.

Another contribution of this work is that *C. difficile* spores seem to directly interact with the phagosome’s membrane of Raw 264.7 cells and presumably induce membrane disruption and cell death. The precise nature of this interaction is unclear but is likely to involve interactions between the spore surface proteins and the phagosome’s membrane and/or membrane embedded proteins. It is also unclear if these interactions might have any implication in the survival of *C. difficile* spores during infection of macrophages or in macrophage death, but these interactions have been shown to occur between *C. perfringens* vegetative cells and the membrane phagosome of J779 cells [35], as well as between the hair-like nap of *B. anthracis* spores and the phagosome’s membrane [21]. Our current results demonstrated that during extended incubation, *C. difficile* spores were able to induce permanent damage to Raw 264.7 cells leading to disruption of the plasma membrane and loss of viability of Raw 264.7 cells as judged by the penetration of ethidium homodimer-1 and trypan blue. Despite the membrane damage, Raw 264.7 cells retained significant esterase activity as shown by flurescence of calcein dye. In conclusion, our work provides evidence that *C. difficile* spores are recognized by phagocytic cells but remain impermeable to attacks by their antimicrobial components and are able to exert cytotoxic effects to Raw 264.7 cells. The implications of these findings clearly suggest that *C. difficile* spores are able to subvert attacks of host’s immune system avoiding spore clearance and persist in the colonic tract.

## References

[1] Deneve C, Janoir C, Poilane I, Fantinato C, Collignon A (2009) New trends in *Clostridium difficile* virulence and pathogenesis. Int J Antimicrob Agents 33 Suppl 1: S24–28. 19303565 10.1016/S0924-8579(09)70012-3

[2] Rupnik M, Wilcox MH, Gerding DN (2009) *Clostridium difficile* infection: new developments in epidemiology and pathogenesis. Nat Rev Microbiol 7: 526–536. 19528959 10.1038/nrmicro2164

[3] Goulding D, Thompson H, Emerson J, Fairweather NF, Dougan G, et al (2009) Distinctive profiles of infection and pathology in hamsters infected with *Clostridium difficile* strains 630 and B1. Infect Immun 77: 5478–5485. 19752031 10.1128/IAI.00551-09 PMC2786451

[4] Sethi AK, Al-Nassir WN, Nerandzic MM, Bobulsky GS, Donskey CJ (2010) Persistence of skin contamination and environmental shedding of *Clostridium difficile* during and after treatment of *C. difficile* infection. Infect Control Hosp Epidemiol 31: 21–27. 19929371 10.1086/649016

[5] Paredes-Sabja D, Sarker MR (2012) Adherence of *Clostridium difficile* spores to Caco-2 cells in culture. J Med Microbiol In Press. 10.1099/jmm.0.043687-0 22595914

[6] Baines SD, O’Connor R, Saxton K, Freeman J, Wilcox MH (2009) Activity of vancomycin against epidemic *Clostridium difficile* strains in a human gut model. J Antimicrob Chemother 63: 520–525. 19112083 10.1093/jac/dkn502

[7] Sarker MR, Paredes-Sabja D (2012) Molecular basis of early stages of *Clostridium difficile* infection: germination and colonization. Future Microbiol In Press. 10.2217/fmb.12.64 22913353

[8] Sorg JA, Sonenshein AL (2008) Bile salts and glycine as co-germinants for *Clostridium difficile* spores. J Bacteriol 190: 2505–2512. 18245298 10.1128/JB.01765-07 PMC2293200

[9] Sorg JA, Sonenshein AL (2009) Chenodeoxycholate is an inhibitor of *Clostridium difficile* spore germination. J Bacteriol 191: 1115–1117. 19060152 10.1128/JB.01260-08 PMC2632082

[10] Wheeldon LJ, Worthington T, Lambert PA (2011) Histidine acts as a co-germinant with glycine and taurocholate for *Clostridium difficile* spores. J Appl Microbiol. 10.1111/j.1365-2672.2011.04953.x 21261795

[11] Hasegawa M, Yamazaki T, Kamada N, Tawaratsumida K, Kim YG, et al (2011) Nucleotide-binding oligomerization domain 1 mediates recognition of *Clostridium difficile* and induces neutrophil recruitment and protection against the pathogen. J Immunol 186: 4872–4880. 21411735 10.4049/jimmunol.1003761

[12] Ryan A, Lynch M, Smith SM, Amu S, Nel HJ, et al (2011) A role for TLR4 in *Clostridium difficile* infection and the recognition of surface layer proteins. PLoS Pathog 7: e1002076. 21738466 10.1371/journal.ppat.1002076 PMC3128122

[13] Paredes-Sabja D, Sarker MR (2012) Interactions between *Clostridium perfringens* spores and Raw 264.7 macrophages. Anaerobe 18: 148–156. 22209938 10.1016/j.anaerobe.2011.12.019

[14] Paredes-Sabja D, Sarker N, Setlow B, Setlow P, Sarker MR (2008) Roles of DacB and Spm proteins in *Clostridium perfringens* spore resistance to moist heat, chemicals and UV radiation. Appl Environ Microbiol 74: 3730–3738. 18441110 10.1128/AEM.00169-08 PMC2446547

[15] Raju D, Waters M, Setlow P, Sarker MR (2006) Investigating the role of small, acid-soluble spore proteins (SASPs) in the resistance of *Clostridium perfringens* spores to heat. BMC Microbiol 6: 50. 16759397 10.1186/1471-2180-6-50 PMC1501028

[16] Raju D, Setlow P, Sarker MR (2007) Antisense-RNA-mediated decreased synthesis of small, acid-soluble spore proteins leads to decreased resistance of *Clostridium perfringens* spores to moist heat and UV radiation. Appl Environ Microbiol 73: 2048–2053. 17259355 10.1128/AEM.02500-06 PMC1855649

[17] Paredes-Sabja D, Raju D, Torres JA, Sarker MR (2008) Role of small, acid-soluble spore proteins in the resistance of *Clostridium perfringens* spores to chemicals. Int J Food Microbiol 122: 333–335. 18221812 10.1016/j.ijfoodmicro.2007.12.006

[18] Paredes-Sabja D, Setlow B, Setlow P, Sarker MR (2008) Characterization of *Clostridium perfringens* spores that lack SpoVA proteins and dipicolinic acid. J Bacteriol 190: 4648–4659. 18469104 10.1128/JB.00325-08 PMC2446781

[19] Guidi-Rontani C, Pereira Y, Ruffie S, Sirard JC, Weber-Levy M, et al (1999) Identification and characterization of a germination operon on the virulence plasmid pXO1 of *Bacillus anthracis* . Mol Microbiol 33: 407–414. 10411756 10.1046/j.1365-2958.1999.01485.x

[20] Guidi-Rontani C, Weber-Levy M, Labruyere E, Mock M (1999) Germination of *Bacillus anthracis* spores within alveolar macrophages. Mol Microbiol 31: 9–17. 9987105 10.1046/j.1365-2958.1999.01137.x

[21] Guidi-Rontani C, Levy M, Ohayon H, Mock M (2001) Fate of germinated *Bacillus anthracis* spores in primary murine macrophages. Mol Microbiol 42: 931–938. 11737637 10.1046/j.1365-2958.2001.02695.x

[22] Kang TJ, Fenton MJ, Weiner MA, Hibbs S, Basu S, et al (2005) Murine macrophages kill the vegetative form of *Bacillus anthracis* . Infect Immun 73: 7495–7501. 16239551 10.1128/IAI.73.11.7495-7501.2005 PMC1273904

[23] Wust J, Sullivan NM, Hardegger U, Wilkins TD (1982) Investigation of an outbreak of antibiotic-associated colitis by various typing methods. J Clin Microbiol 16: 1096–1101. 7161375 10.1128/jcm.16.6.1096-1101.1982 PMC272546

[24] Paredes-Sabja D, Sarker MR (2011) Germination response of spores of the pathoenic bacetrium *Clostridium perfringens* and *Clostridium difficile* to cultured human epithelial cells. Anaerobe 17: 78–84. 21315167 10.1016/j.anaerobe.2011.02.001

[25] McEllistrem MC, Carman RJ, Gerding DN, Genheimer CW, Zheng L (2005) A hospital outbreak of *Clostridium difficile* disease associated with isolates carrying binary toxin genes. Clin Infect Dis 40: 265–272. 15655746 10.1086/427113

[26] Pelczar PL, Igarashi T, Setlow B, Setlow P (2007) Role of GerD in germination of *Bacillus subtilis* spores. J Bacteriol 189: 1090–1098. 17122337 10.1128/JB.01606-06 PMC1797312

[27] Nicholson WL, Setlow P (1990) *Sporulation, germination and outgrowth* In: Harwood CR, Cutting SM, editors. Molecular biological methods for *Bacillus*. Chichester: John Wiley and Sons. 391–450.

[28] May JA, Ratan H, Glenn JR, Losche W, Spangenberg P, et al (1998) GPIIb-IIIa antagonists cause rapid disaggregation of platelets pre-treated with cytochalasin D. Evidence that the stability of platelet aggregates depends on normal cytoskeletal assembly. Platelets 9: 227–232. 16793707 10.1080/09537109876744

[29] Allen LA, Aderem A (1996) Molecular definition of distinct cytoskeletal structures involved in complement- and Fc receptor-mediated phagocytosis in macrophages. J Exp Med 184: 627–637. 8760816 10.1084/jem.184.2.627 PMC2192718

[30] Henriques AO, Moran CP Jr (2007) Structure, assembly, and function of the spore surface layers. Annu Rev Microbiol 61: 555–588. 18035610 10.1146/annurev.micro.61.080706.093224

[31] Lawley TD, Croucher NJ, Yu L, Clare S, Sebaihia M, et al (2009) Proteomic and genomic characterization of highly infectious *Clostridium difficile* 630 spores. J Bacteriol 191: 5377–5386. 19542279 10.1128/JB.00597-09 PMC2725610

[32] Horwitz MA (1984) Phagocytosis of the Legionnaires’ disease bacterium (Legionella pneumophila) occurs by a novel mechanism: engulfment within a pseudopod coil. Cell 36: 27–33. 6692469 10.1016/0092-8674(84)90070-9

[33] Groves E, Dart AE, Covarelli V, Caron E (2008) Molecular mechanisms of phagocytic uptake in mammalian cells. Cellular and molecular life sciences : CMLS 65: 1957–1976. 18322649 10.1007/s00018-008-7578-4 PMC11131742

[34] Sebaihia M, Wren BW, Mullany P, Fairweather NF, Minton N, et al (2006) The multidrug-resistant human pathogen *Clostridium difficile* has a highly mobile, mosaic genome. Nat Genet 38: 779–786. 16804543 10.1038/ng1830

[35] O’Brien DK, Melville SB (2000) The anaerobic pathogen *Clostridium perfringens* can escape the phagosome of macrophages under aerobic conditions. Cell Microbiol 2: 505–519. 11207604 10.1046/j.1462-5822.2000.00074.x

[36] Paredes-Sabja D, Sarker MR (2011) Host serum factor triggers germination of *Clostridium perfringens* spores lacking cortex hydrolysis machinery. J Med Microbiol In Press. 10.1099/jmm.0.031575-0 21799201

[37] Giebel JD, Carr KA, Anderson EC, Hanna PC (2009) The germination-specific lytic enzymes SleB, CwlJ1, and CwlJ2 each contribute to *Bacillus anthracis* spore germination and virulence. J Bacteriol 191: 5569–5576. 19581364 10.1128/JB.00408-09 PMC2737968

[38] Setlow P (2003) Spore germination. Curr Opin Microbiol 6: 550–556. 14662349 10.1016/j.mib.2003.10.001

[39] Flannagan RS, Jaumouille V, Grinstein S (2012) The cell biology of phagocytosis. Annual review of pathology 7: 61–98. 10.1146/annurev-pathol-011811-132445 21910624

[40] Gros P, Milder FJ, Janssen BJ (2008) Complement driven by conformational changes. Nat Rev Immunol 8: 48–58. 18064050 10.1038/nri2231

[41] Chung MC, Tonry JH, Narayanan A, Manes NP, Mackie RS, et al (2011) *Bacillus anthracis* interacts with plasmin(ogen) to evade C3b-dependent innate immunity. PLoS One 6: e18119. 21464960 10.1371/journal.pone.0018119 PMC3064659

[42] Mueller-Ortiz SL, Wanger AR, Norris SJ (2001) *Mycobacterial* protein HbhA binds human complement component C3. Infect Immun 69: 7501–7511. 11705926 10.1128/IAI.69.12.7501-7511.2001 PMC98840

[43] Lahteenmaki K, Edelman S, Korhonen TK (2005) Bacterial metastasis: the host plasminogen system in bacterial invasion. Trends Microbiol 13: 79–85. 15680767 10.1016/j.tim.2004.12.003

[44] Okumura Y, Yano M, Murakami M, Mori S, Towatari T, et al (1999) The extracellular processing of HIV-1 envelope glycoprotein gp160 by human plasmin. FEBS Lett 442: 39–42. 9923600 10.1016/s0014-5793(98)01612-3

[45] Stie J, Bruni G, Fox D (2009) Surface-associated plasminogen binding of *Cryptococcus neoformans* promotes extracellular matrix invasion. PLoS One 4: e5780. 19492051 10.1371/journal.pone.0005780 PMC2685986

[46] Hu H, Emerson J, Aronson AI (2007) Factors involved in the germination and inactivation of *Bacillus anthracis* spores in murine primary macrophages. FEMS Microbiol Lett 272: 245–250. 17521404 10.1111/j.1574-6968.2007.00766.x

[47] Lawley TD, Clare S, Walker AW, Goulding D, Stabler RA, et al (2009) Antibiotic treatment of *Clostridium difficile* carrier mice triggers a supershedder state, spore-mediated transmission, and severe disease in immunocompromised hosts. Infect Immun 77: 3661–3669. 19564382 10.1128/IAI.00558-09 PMC2737984

